# Amphid sensory neurons of *Caenorhabditis elegans* orchestrate its survival from infection with broad classes of pathogens

**DOI:** 10.26508/lsa.202301949

**Published:** 2023-05-31

**Authors:** Siddharth R Venkatesh, Anjali Gupta, Varsha Singh

**Affiliations:** 1 Department of Developmental Biology & Genetics, Indian Institute of Science, Bangalore, INDIA; 2 Center for Biosystems, Science and Engineering, Indian Institute of Science, Bangalore, INDIA

## Abstract

The survival of a host during infection relies on its ability to rapidly sense the invading pathogen and mount an appropriate response. The bacterivorous nematode *Caenorhabditis elegans* lacks most of the traditional pattern recognition mechanisms. In this study, we hypothesized that the 12 pairs of amphid sensory neurons in the heads of worms provide sensing capability and thus affect survival during infection. We tested animals lacking amphid neurons to three major classes of pathogens, namely—a Gram-negative bacterium *Pseudomonas aeruginosa*, a Gram-positive bacterium *Enterococcus faecalis*, and a pathogenic yeast *Cryptococcus neoformans*. By using individual neuronal ablation lines or mutants lacking specific neurons, we demonstrate that some neurons broadly suppress the survival of the host and colonization of all pathogens, whereas other amphid neurons differentially regulate host survival during infection. We also show that the roles of some of these neurons are pathogen-specific, as seen with the AWB odor sensory neurons that promote survival only during infections with *P*. *aeruginosa*. Overall, our study reveals broad and specific roles for amphid neurons during infections.

## Abstract (precis)

This study shows that the nervous system of the bacterivorous nematode *Caenorhabditis elegans* coordinates pathogen-specific and broad immune responses during infection.

## Graphical Abstract

**Figure:**
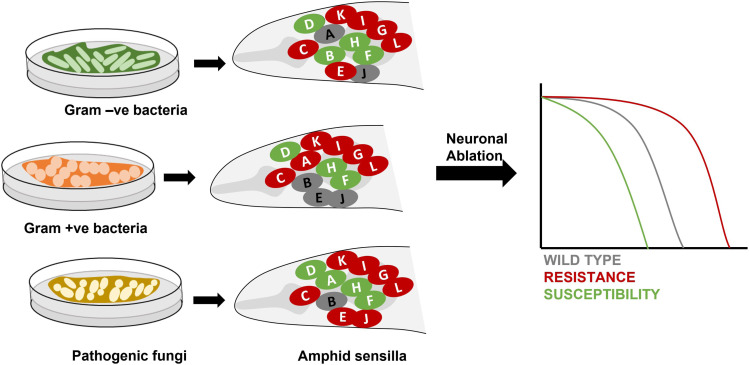

## Introduction

Adaptation to the changing environment is a major determinant of organismal survival (Rosalino et al, 2014). Rapid and accurate sensing of invading pathogens is imperative for host survival. Pathogen sensing in most invertebrates and all vertebrates is achieved via the detection of pathogen-associated molecular patterns (PAMPs) by pattern recognition receptors (PRRs) of the host (Akira et al, 2006; Hansen et al, 2011). The PAMPS such as the lipopolysaccharide (Park & Lee, 2013) and O-antigen in Gram-negative bacteria, the peptidoglycan and lipoteichoic acid in Gram-positive bacteria (Wolf & Underhill, 2018), flagellin in all flagellated bacteria (Uematsu et al, 2006), and mannoproteins, beta-glucans, and chitin from yeast (Sorrell & Chen, 2009) allow the host to distinguish between major classes of pathogens and facilitate an appropriate innate immune response. Some of the PRRs include membrane-associated toll-like receptors (TLRs) and soluble receptors, such as the nod-like receptors (NLRs), RIG-I-like receptors (RLRs), AIM-like receptors (ALRs), and c-type lectins (Hansen et al, 2011). Many invertebrates, such as *Caenorhabditis elegans*, have a reduced repertoire of canonical PRRs suggesting they may have alternate mechanisms of pathogen sensing.

In *C. elegans*, a free-living, bacterivorous nematode, the only TLR homolog called TOL-1 is nonessential during most infections except those with *Salmonella enterica* (Tenor & Aballay, 2008). An RLR homolog, DHR-1, is involved in sensing intracellular pathogens such as the Orsay virus (Sowa et al, 2020), whereas some c-type lectins such as CLEC-39 and CLEC-49 mediate survival on pathogenic bacteria *Serratia marcescens* (Miltsch et al, 2014). Despite the lack of conserved PRRs, *C. elegans* is capable of mounting pathogen-specific immune responses (Irazoqui et al, 2010; Engelmann et al, 2011; Dasgupta et al, 2020). Several lines of evidence suggest that G-protein-coupled receptors (GPCRs) may serve as non-canonical PRRs in *C. elegans*. Of the nearly 1,300 putative GPCRs (Thomas & Robertson, 2008) encoded in its genome, some play crucial roles during infection by sensing bacterial PAMPs and host damage-associated molecular patterns (DAMPs) (Venkatesh & Singh, 2021). The OLRN-1, OCTR-1, and NPR-8 GPCRs are known to repress host immunity, whereas NPR-1 and FSHR-1 are required to initiate protective immune responses (Styer et al, 2008; Powell et al, 2009; Sun et al, 2011; Miller et al, 2015; Sellegounder et al, 2019; Foster et al, 2020; Kim & Sieburth, 2020). Other than FSHR-1 and DCAR-1, most of the immune-modulating GPCRs are expressed predominantly in the nervous system. These results suggest that GPCRs and sensory neurons of *C. elegans* have important roles in organismal survival.

The *C. elegans* nervous system comprises 302 neurons, of which 12 pairs of neurons in the head form the amphid sensilla. (White et al, 1986). The amphid neurons include the odor sensory neurons AWA, AWB, and AWC; the thermosensory neuron AFD; the nociceptive neuron ASH; and chemosensory neurons ASE, ASG, ASI, ASK, ADF, ASJ, and ADL. These neurons sense a variety of cues, including salts, detergents, pH, pheromones, bacterial volatiles, and secondary metabolites. Some of the physiological responses regulated by the amphid neurons include the nociceptive aversion to noxious stimuli such as heavy metals and detergents regulated by ASH neurons (Guo et al, 2015), the sexual attraction in males mediated by AWA, AWB, and ASK neurons (White et al, 2007; White & Jorgensen, 2012; Wan et al, 2019), regulation of adult lifespan (Alcedo & Kenyon, 2004), and olfaction-mediated chemotaxis by AWA, AWB, and AWC neurons. The ASG neurons regulate a trade-off between lifespan and immunity (Otarigho & Aballay, 2021). Recent evidence shows that the amphid neurons might also be involved in sensing pathogens and their components. The ASJ and ASI neurons are involved in sensing and responding to pyochelin and pyoverdine, whereas the AWB neuron detects 1-undecene produced by *Pseudomonas aeruginosa* (Meisel et al, 2014; Prakash et al, 2021). Several immunomodulatory GPCRs, such as OLRN-1, OCTR-1, and NPR-8 are expressed and functional in these amphid neurons. These independent studies suggest that specific amphid neurons may have essential functions during infection.

In this study, we investigated whether neurons in the amphid sensilla regulate host survival during infection in *C. elegans*. We subjected worms lacking individual neurons to infection with three classes of pathogens—a Gram-positive bacterium, a Gram-negative bacterium, and a pathogenic yeast. We found that the amphid sensory neurons indeed play pivotal roles in the survival of worms during infection. We observed that the altered survival of sensory mutants is consistent with altered bacterial loads in the infected animals. Altogether, our study shows that some amphid neurons have pathogen-specific roles during infection, whereas others have global regulatory roles.

## Results

### *C. elegans* odor sensory neurons modulate host survival during infection

**Figure 1.**
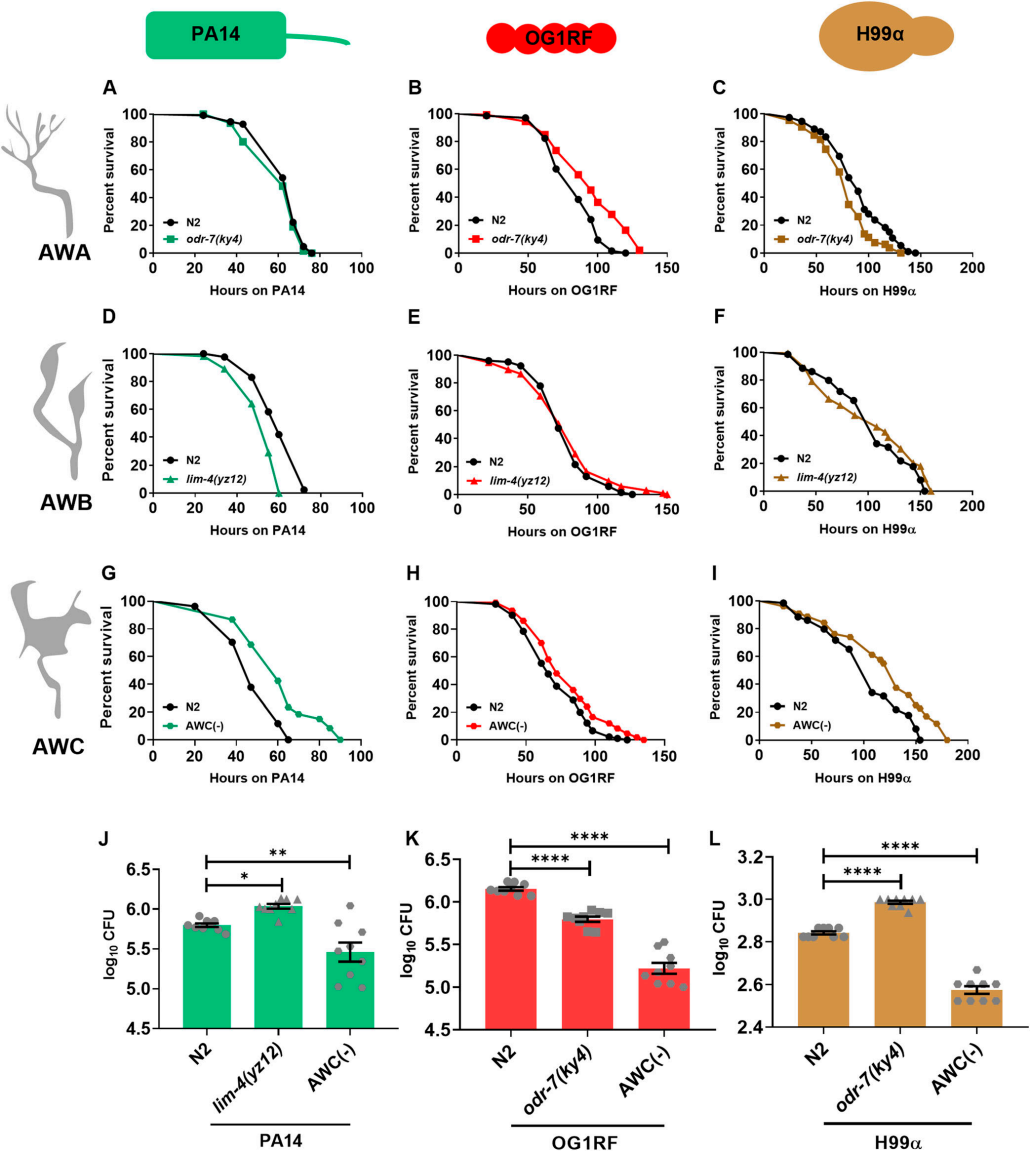
Odor sensory neurons regulate survival during infection. **(A, B, C)** Kaplan–Meier survival curves for N2 and *odr-7(ky4)* on (A) *P. aeruginosa* PA14 (*P* = 0.126), (B) *E. faecalis* OG1RF (*P* < 0.0001), and (C) *C. neoformans* H99α (*P* = 0.0011). **(D, E, F)** Kaplan–Meier survival curves for N2 and *lim-4(yz12)* on (D) PA14 (*P* < 0.0001), (E) OG1RF (*P* = 0.5), and (F) H99α (*P* = 0.46). **(G, H, I)** Kaplan–Meier survival curves for N2 and AWC(−) on (G) PA14 (*P* < 0.0001), (H) OG1RF (*P* < 0.0001), and (I) H99α (*P* < 0.0001). **(J, K, L)** Intestinal pathogen load quantified as CFU 24 h postinfection with (J) PA14, (K) OG1RF, and (L) H99α. Significance was calculated using post-hoc Dunnett’s test. Non-significant, *P* > 0.05; *, *P* < 0.05; **, *P* < 0.01. The error bar signifies the SEM. Source data are available for this figure.

**Figure S1.**
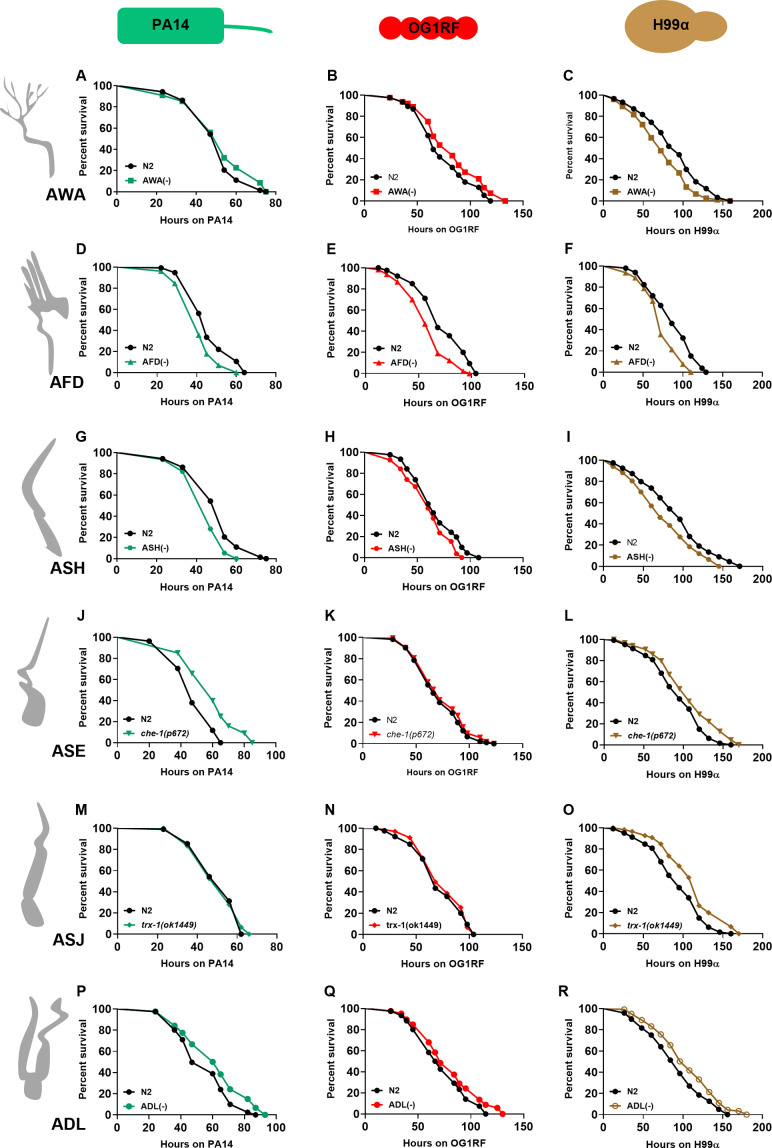
Ablation of amphid sensory neurons affects the survival of worms during infection. **(A, B, C)** Kaplan–Meier survival curves for N2 and AWA(−) on (A) *P. aeruginosa* PA14 (*P* = 0.1288), (B) *E. faecalis* OG1RF (*P* = 0.0111), and (C) *C. neoformans* H99α (*P* = 0.0012). **(D, E, F)** Kaplan–Meier survival curves for N2 and AFD(−) on (D) PA14 (*P* < 0.0001), (E) OG1RF (*P* < 0.0001), and (F) H99α (*P* = 0.0002). **(G, H, I)** Kaplan–Meier survival curves for N2 and ASH(−) on (G) PA14 (*P* < 0.0001), (H) OG1RF (*P* = 0.038), and (I) H99α (*P* = 0.0022). **(J, K, L)** Kaplan–Meier survival curves for N2 and *che-1(p672)* on (J) PA14 (*P* < 0.0001), (K) OG1RF (*P* = 0.3239), and (L) H99α (*P* = 0.0042). **(M, N, O)** Kaplan–Meier survival curves for N2 and *trx-1(ok1449)* on (M) PA14 (*P* = 0.9437), (N) OG1RF (*P* = 0.6336), and (O) H99α (*P* = 0.0001). **(P, Q, R)** Kaplan–Meier survival curves for N2 and ASH(−) on (P) PA14 (*P* = 0.0004), (Q) OG1RF (*P* = 0.0318), and (R) H99α (*P* = 0.0042). Nonsignificant, *P* > 0.05; *, *P* < 0.05; **, *P* < 0.01; ***, *P* < 0.001; ****, *P* < 0.001.

**Figure S2.**
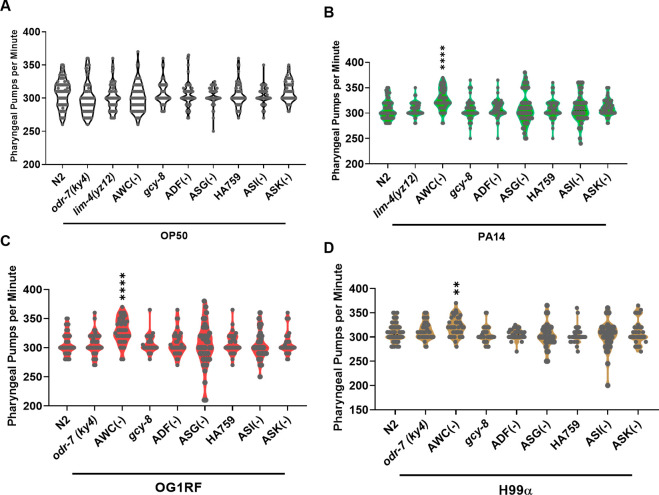
Quantification of pharyngeal pumping. **(A, B, C, D)** Pharyngeal pumping rate of young adults after 12 h on (A) OP50, (B) PA14, (C) OG1RF or (D) H99α. Significance was calculated using post-hoc Dunnett’s test. Nonsignificant, *P* > 0.05; **, *P* < 0.01; ****, *P* < 0.0001. Error bars indicate SEM. Source data are available for this figure.

To understand the role of odor sensory neurons, AWA, AWB, and AWC, in survival, we subjected worms lacking individual pairs of neurons to infection with a Gram-negative bacterium *P. aeruginosa* PA14, a Gram-positive bacterium *Enterococcus faecalis* OG1RF, and a pathogenic yeast *Cryptococcus neoformans* H99α. To study the role of the AWA pair of neurons, we used a mutation in the *odr-7* gene that encodes a nuclear hormone receptor required for the identity of the AWA neurons (Sengupta et al, 1994; Colosimo et al, 2003). The *odr-7* mutants and N2 WT animals were infected with each of the three pathogens, one at a time. The *odr-7* mutants had a survival phenotype similar to that of N2 worms during infection with PA14 (Fig 1A); however, they displayed a slight but significant resistance to OG1RF, and a significant susceptibility to H99α (Fig 1B and C, also see Tables S1 and S2). To confirm these observations, we used a genetic ablation line of AWA neurons by expressing split caspase under the *odr-10* promoter (Prakash et al, 2021). This strain phenocopied the survival phenotype of *odr-7* mutants during infection (Fig S1A–C). We also analyzed the intestinal load of pathogens between the various genotypes of *C. elegans*. The *odr-7* worms displayed a lower intestinal load of OG1RF (Fig 1K) and a higher intestinal load of H99α than N2 worms for each of these pathogens 24 h postinfection (Fig 1L), consistent with their resistance to OG1RF and susceptibility to the pathogenic yeast. Interestingly, the pharyngeal pumping rates of *odr-7* and N2 worms on *Escherichia coli* OP50 and on pathogens were comparable (Fig S2), suggesting that the differences in intestinal pathogen load on OG1RF and H99α were unlikely to be a result of differential pathogen intake.

Table S1. Survival curve statistics.

Table S2. The table summarises the relative percentage of TD50 values for mutants or amphid neuronal ablation lines.

To test the role of AWB odor sensory neurons, we used worms with a mutation in the *lim-4* homeobox transcription factor that renders AWB nonfunctional (Sagasti et al, 1999). We observed that *lim-4* mutants were more susceptible to PA14 than N2 worms, consistent with a previous report (Prakash et al, 2021), whereas they had WT survival phenotype upon OG1RF or H99α infection (Fig 1D–F). The *lim-4* mutants had a higher intestinal load of PA14 compared with N2 worms (Fig 1J) despite WT pharyngeal pumping rates (Fig S2).

We investigated the effect of AWC odor sensory neurons on survival during infection using AWC ablation worms, termed AWC(−), generated via the expression of a split caspase (Beverly et al, 2011). The AWC(−) worms consistently displayed enhanced resistance to all three pathogens (Fig 1G–I). The AWC(−) worms had an elevated pharyngeal pumping rate (Fig S2); however, they still had a lower pathogen load compared with infected N2 worms (Fig 1J–L), suggesting that these worms were better at clearing pathogens despite enhanced ingestion.

Taken together, our data suggest that AWB neurons have a unique role in survival on *P*. *aeruginosa*, the AWA neurons are uniquely but differentially involved in survival on *E*. *faecalis* and *C*. *neoformans*, whereas the AWC neurons suppress survival during all the tested infections.

### Thermosensory, serotonergic, and nociceptive amphid neurons of *C. elegans* are positive regulators of survival during infection

**Figure 2.**
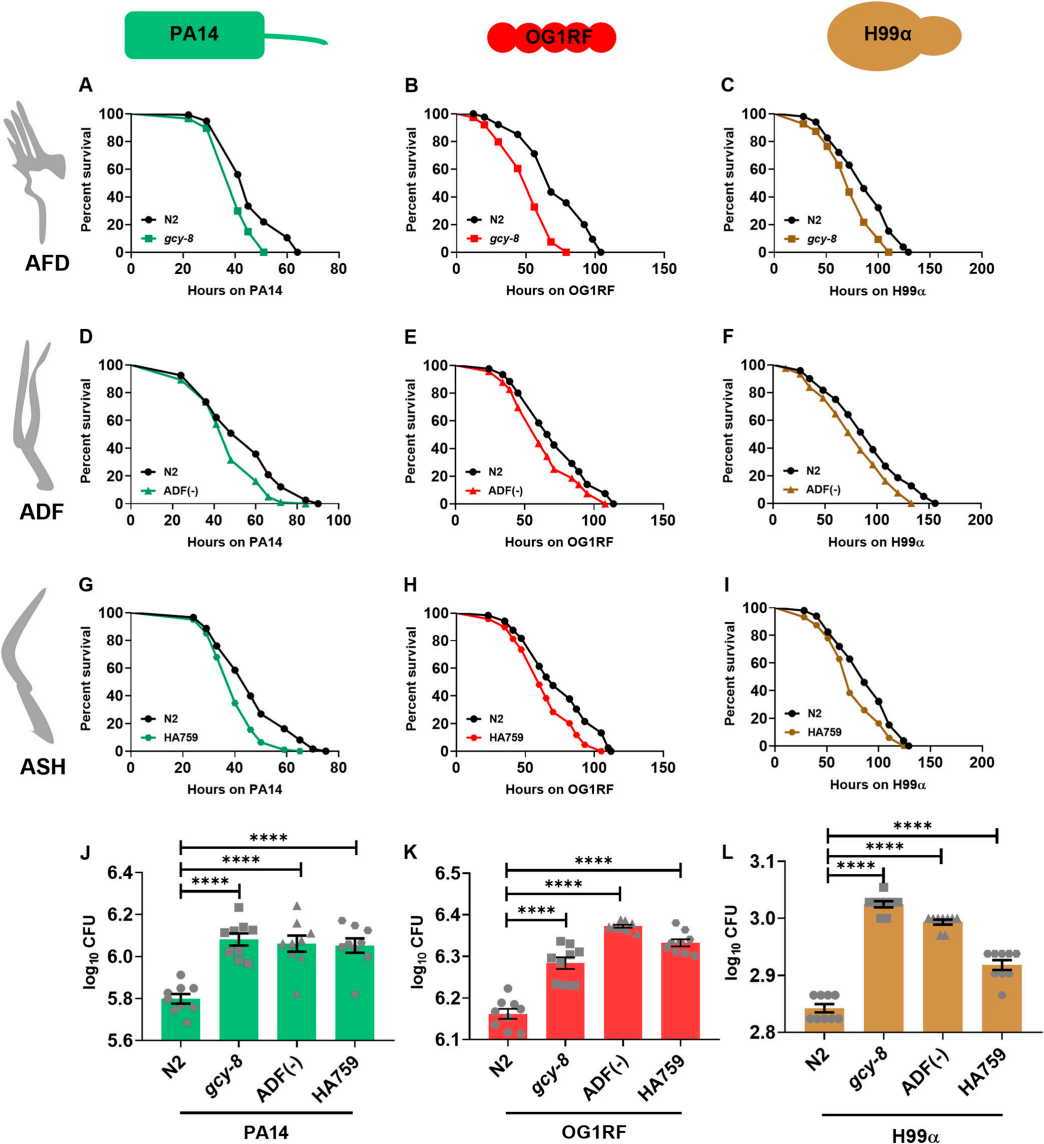
Thermosensory, nociceptive, and serotonergic neurons positively regulate survival during infection. **(A, B, C)** Kaplan–Meier survival curves for N2 and *gcy-8(oy44)* on (A) PA14 (*P* < 0.0001), (B) OG1RF (*P* < 0.0001), and (C) H99α (*P* = 0.0002). **(D, E, F)** Kaplan–Meier survival curves for N2 and ADF(−) on (D) PA14 (*P* < 0.0001), (E) OG1RF (*P* < 0.0001), and (F) H99α (*P* < 0.0001). **(G, H, I)** Kaplan–Meier survival curves for N2 and HA759 on (G) PA14 (*P* < 0.001), (H) OG1RF (*P* < 0.01), and (I) H99α (*P* = 0.004). **(J, K, L)** Intestinal pathogen loads were quantified as CFU 24 h post infection with (J) PA14, (K) OG1RF, and (L) H99α. Significance was calculated using post-hoc Dunnett’s test. Non-significant, *P* > 0.05; ****, *P* < 0.0001. The error bar signifies the SEM. Source data are available for this figure.

We tested the survival regulatory roles of amphid neurons constituting the thermosensory (AFD), polymodal (ASH), and serotonergic (ADF) neurons. To investigate if AFD neurons, the principal thermosensory neurons in worms, mediate host survival, we used a split caspase-mediated ablation line termed AFD(−) (Wang et al, 2013). We also used a mutation in the *gcy-8* gene encoding an AFD-specific guanylyl cyclase required for thermosensation (Wang et al, 2013). We observed that the *gcy-8* mutants were sensitive to infection with all three pathogens (Fig 2A–C). AFD(−) worms also showed enhanced susceptibility to infection (Fig S1D–F). Despite WT pharyngeal pumping rates in these worms on pathogens (Fig S2), they exhibited significantly higher pathogen load compared with N2 controls (Fig 2J–L), consistent with their susceptibility to infection. Using a split caspase ablation line for ADF neurons, termed ADF(−), we found that they were also susceptible to infection with all three pathogens (Fig 2D–F). ADF(−) worms also had a higher intestinal load of pathogens than N2 worms (Fig 2J–L), consistent with their survival phenotypes. To test if the nociceptive chemosensory neurons, ASH neurons, are also involved in modulating survival during infection, we used two strains, a split caspase ablation line, termed ASH(−) (Prakash et al, 2021), and HA759 worms, where the ASH neurons were rendered nonfunctional because of the expression of polyglutamine (polyQ) resulting in cell death (Faber et al, 1999). Upon infection with any of the three pathogens, HA759 worms were more susceptible than N2 worms (Fig 2G–I). We also observed susceptibility to infection in ASH(−) worms (Fig S1G–I). We found that HA759 worms also had an increased pathogen load than N2 worms (Fig 2J–L), consistent with their enhanced susceptibility to infections.

In all, we show that three pairs of amphid neurons, thermosensory neuron AFD, serotonergic neuron ADF, and nociceptive neuron ASH, are essential for host survival during infection with three distinct classes of pathogens.

### ASG, ASI, and ASK chemosensory neurons are negative regulators of survival during infection

**Figure 3.**
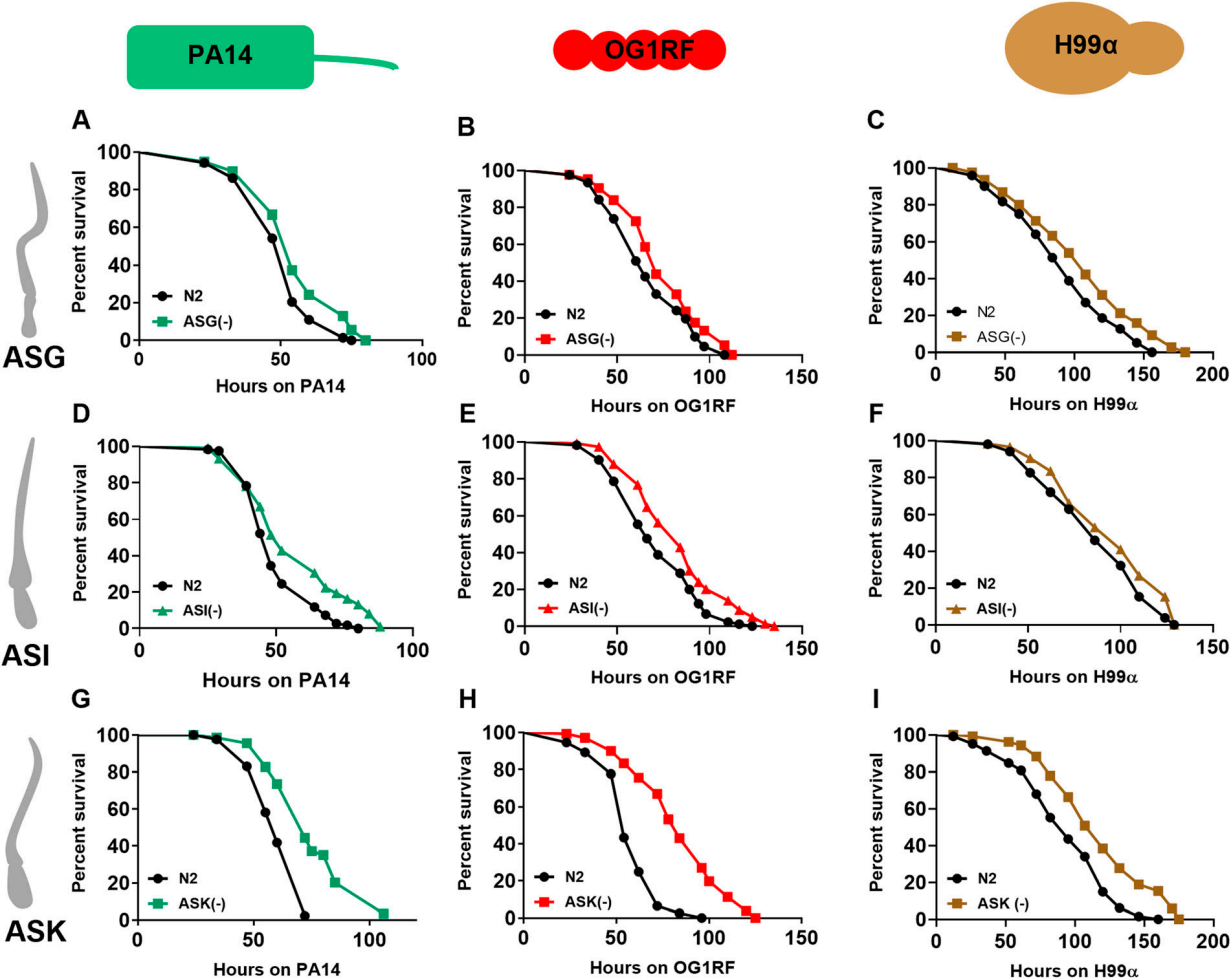
ASG, ASI, and ASK neurons suppress survival during infection. **(A, B, C)** Kaplan–Meier survival curves for N2 and ASG(−) on (A) PA14 (*P* = 0.003), (B) OG1RF (*P* = 0.005), and (C) H99α (*P* = 0.002). **(D, E, F)** Kaplan–Meier survival curves for N2 and ASI(−) on (D) PA14 (*P* = 0.0001), (E) OG1RF (*P* = 0.0004), and (F) H99α (*P* = 0.04). **(G, H, I)** Kaplan–Meier survival curves for N2 and ASK(−) on (G) PA14 (*P* < 0.0001), (H) OG1RF (*P* < 0.0001), and (I) H99α (*P* < 0.0001).

**Figure S3.**
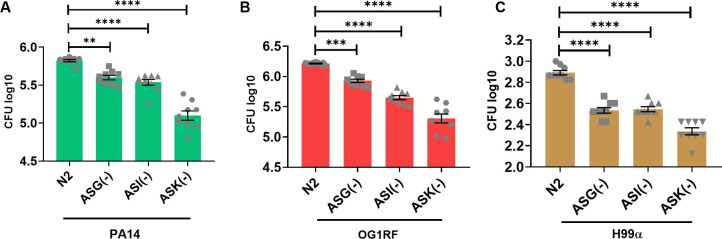
Ablation of ASG, ASI, and ASK neurons alleviates intestinal pathogen load. **(A, B, C)** Intestinal pathogen loads of N2, ASG(−), ASI(−), and ASK(−) quantified as CFU 24 h postinfection with (A) PA14, (B) OG1RF, and (C) H99α. Significance was calculated using post-hoc Dunnett’s test. **, *P* < 0.01; ***, *P* < 0.001; ****, *P* < 0.0001. Source data are available for this figure.

We investigated the effect of remaining amphid neurons on survival during infection. To investigate the effect of ASG neurons on survival, we used a split caspase ablation line, ASG(−) worms, in infection assays with the three test pathogens. The ASG(−) worms were more resistant than N2 worms on all three pathogens (Fig 3A–C). Similarly, we observed that ASI(−) worms (Beverly et al, 2011), lacking functional ASI neurons, were also resistant to all three pathogens (Fig 3D–F). We used a mouse caspase-mediated ablation of ASK, ASK(−) worms, in infection assays (Srinivasan et al, 2012). The ASK(−) worms displayed enhanced resistance to all three pathogens (Fig 3G–I). The enhanced resistance phenotype of ASK(−) was more profound than that displayed by AWC(−), ASG(−) or ASI(−) worms (Tables S1 and S2). Consistent with the survival phenotype, infected ASG(−), ASI(−), and ASK(−) worms showed lowered loads of PA14 (Fig S3A), OG1RF (Fig S3B), and H99α (Fig S3C) in the intestine compared with infected N2 worms. Our results demonstrate a hitherto unknown role of ASK neurons in the global repression of survival during infection.

We also analyzed other chemosensory neurons in the amphid sensilla. We found a differential effect of ASE neurons on survival in worms using *che-1* mutation as a proxy for nonfunctional ASE chemosensory neurons (Uchida et al, 2003). The *che-1* mutants showed enhanced susceptibility to PA14 and H99α but WT survival on OG1RF (Fig S1J–L). A mutant of *trx-1*, required for ASJ neuron functioning (Miranda-Vizuete et al, 2006; Fierro-Gonzalez et al, 2011) had WT survival phenotype during infection with either PA14 or OG1RF but showed resistance to infection with H99α (Fig S1M–O). In addition to ASG, ASI, and ASK, we also found the ADL neurons to have a small but significant suppressive effect on survival during infections because the ablation of these neurons, ADL(−) worms, leads to enhanced resistance to all three classes of pathogens (Fig S1P–R).

**Figure 4.**
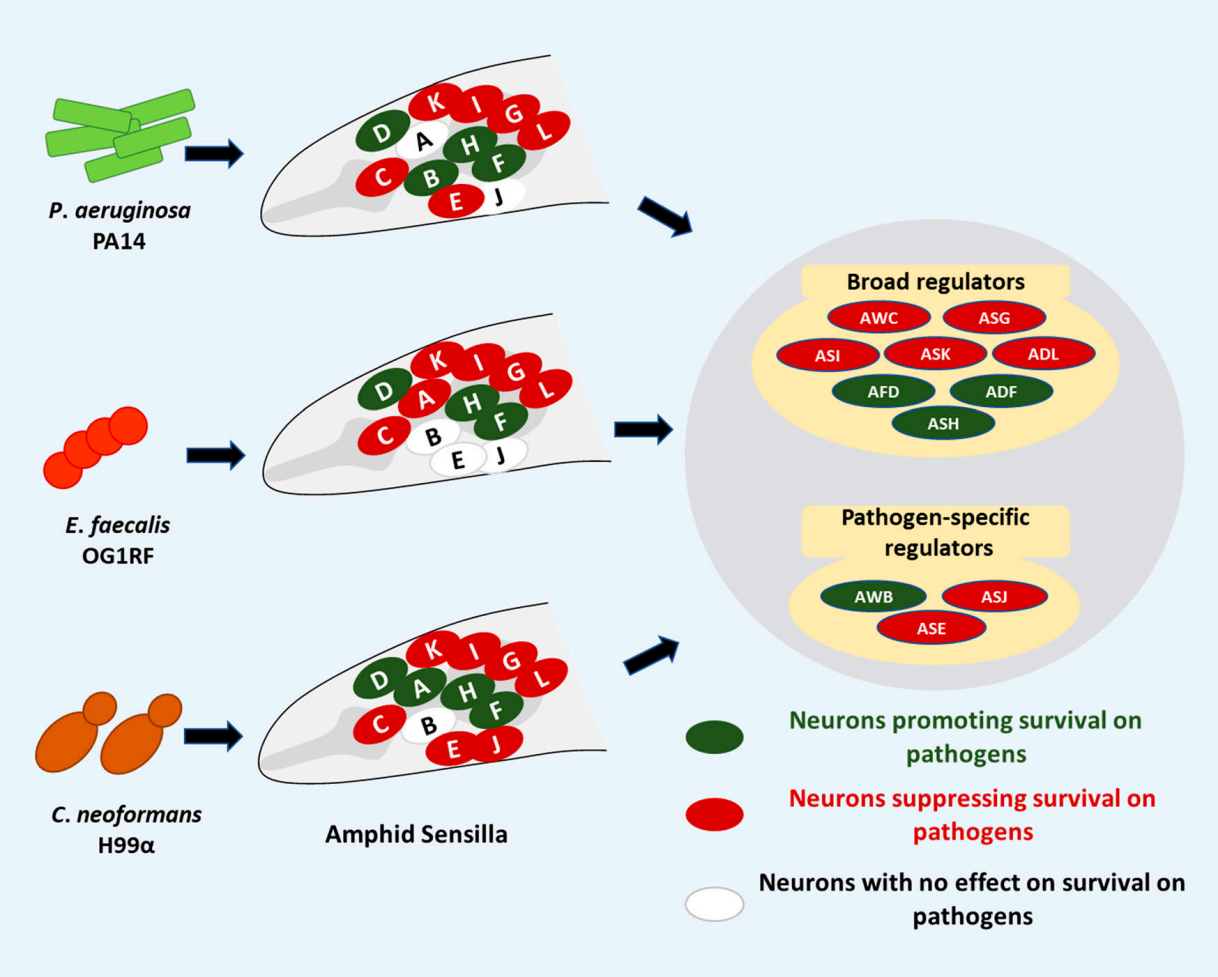
Regulation of survival of infected *C. elegans* by neurons in its amphid sensilla. Several neurons, shown in red, suppress survival to Gram-negative bacteria, Gram-positive bacteria, and pathogenic yeast. Other neurons, shown in green, promote survival during infection with a broad range of pathogens. Other amphid neurons, AWB, ASJ, and ASE, have pathogen-specific roles.

Based on the analyses of *C. elegans* survival on three classes of pathogens, namely Gram-negative bacteria, Gram-positive bacteria, and pathogenic yeast, we build the first model for broad regulation of survival by the amphid sensilla in *C. elegans*. As shown in Table S2 and Fig 4, five pairs of amphid sensory neurons, shown in red, suppress survival. There are also three pairs of neurons, shown in green, that promote survival during infection. These provide means to activate protective immunity or dampen runaway inflammation. We also found evidence for pathogen-specific regulators—AWA for *E*. *faecalis* and *C*. *neoformans*, AWB for *P*. *aeruginosa*, ASE for *P. aeruginosa* and *C. neoformans,* and ASJ for *C*. *neoformans*.

## Discussion

In this study, we examined the roles of the 12 amphid sensory neurons during infection. We show that three amphid neurons, AFD, ADF, and ASH, are essential for promoting survival during infection with all three classes of pathogens, whereas five neurons, AWC, ASG, ASI, ASK, and ADL, suppress survival on the same pathogens. The remaining four neurons (AWA, AWB, ASE, and ASJ) play pathogen-specific survival-modulatory roles. The survival phenotypes were consistent with intestinal pathogen load, suggesting that the neurons were controlling the antimicrobial defences of the host. Overall, this study establishes the broad role of the largest sensory organ, amphid sensilla of *C. elegans*, in the regulation of survival mechanisms in an animal which is constantly exposed to pathogenic microbes in its natural habitat.

The AWB neuron was found to be essential only during infections with *P*. *aeruginosa*, whereas it was dispensable during infections with *E*. *faecalis* and *C*. neoformans. A previous study from our group has shown that the AWB neurons sense 1-undecene, a PAMP produced by *P*. *aeruginosa*, and trigger an aversive response and a *P. aeruginosa*-specific immune response (Prakash et al, 2021). This volatile is not reported to be produced by other Gram-negative bacteria, Gram-positive bacteria, and yeast. Other aversive volatiles have not so far been reported from Gram-positive bacteria and yeast, consistent with the idea that AWB neurons may not have a broad role in survival during infection with them.

Our study provides support for the survival-promoting role of AFD, ADF, and ASH neurons during infection with three pathogen classes. Several protective mechanisms could be initiated by these neurons. The AFD and ADF neurons are known to play important roles in maintaining proteostasis in the endoplasmic reticulum during infection through β-arrestin signaling (Singh & Aballay, 2012). In addition, the AFD thermosensory neurons are also known to mediate the expression of small chaperones that also assist in proteostasis and protect the host from infections with *P*. *aeruginosa* and *E*. *faecalis* (Singh & Aballay, 2006; Prahlad et al, 2008; Morton & Lamitina, 2013). Thus, the AFD neuron might help prolong survival by maintaining proteostasis. ADF neurons are serotonergic, and serotonin signaling through the serotonergic GPCRs is implicated in aversive learning during *P*. *aeruginosa* exposures and in modulating host immune responses (Zhang et al, 2005; Liang et al, 2006; Xie et al, 2013). ASH is a polymodal neuron with the ability to detect a range of noxious stimuli. Our findings suggest that ASH neurons may sense unidentified noxious molecules from the pathogens used in our study. Some of these may be in the form of PAMPs or DAMPs.

We identified AWC, ASG, ASI, ASK, and ADL neurons as broad but nonspecific suppressors of host survival during infection with varying degrees of effects. It was surprising that such a large subset of amphid neurons had a broad role in suppressing survival during infection with pathogens with largely nonoverlapping PAMPs. This suggests that neuronal mechanisms to control runaway immunity or inflammation are important in *C. elegans* as well. The requirement of many neurons also suggests their contextual role in inflammation triggered by various PAMPs or DAMPs. There is some mechanistic evidence for how this might be accomplished. OLRN-1, a GPCR expressed in the AWC neurons, modulates immunity in a cell nonautonomous fashion by repressing intestinal P38 MAPK pathway (Foster et al, 2020), a keystone innate immunity pathway in *C. elegans* for antimicrobial defence. The ASG neurons are known to inhibit immune responses through the repression of two regulators of innate immunity, PMK-1 and ELT-2 GATA transcription factor (Otarigho & Aballay, 2021). The mechanistic basis for the regulation of survival by ASI, ASK, and ADL neurons remains to be studied.

Interestingly, some of the amphid neurons regulate lifespan in *C. elegans* via mechanisms which are not clearly understood (Alcedo & Kenyon, 2004). Laser-mediated ablation of AWA neurons extended lifespan, whereas AWC and ADF ablations had no effect on adult lifespan. In contrast, our study shows that the AWC and ADF neurons act as global regulators of survival on all pathogens, whereas the AWA neurons modulate survival in a pathogen-specific manner. We also show that the ASK neurons that promote the extension of adult lifespan, repress immunity, and host survival during infection. Longevity and immunity have classically shared a strong relationship because of the significant contribution of proteostatic mechanisms, including those of chaperones, on both life span and innate immunity. Trade-offs between immunity and other metabolically expensive traits, such as reproduction, have been identified across living organisms, including plants, invertebrates, and vertebrates (Lozano-Duran & Zipfel, 2015; Fabian et al, 2021). In *C. elegans*, the two are tightly linked in that several immune response genes and pathways, induced upon pathogen exposure, also play crucial roles during cytotoxicity and other stress, thereby affecting lifespan (Kenyon et al, 1993; Lee et al, 2003; Singh & Aballay, 2009). However, there are also studies that delink immunity and lifespan (Amrit et al, 2019). Our study suggests that the effects of amphid neurons are likely to be contextual and dependent on molecular patterns and not necessarily because of the regulation of aging pathways.

The new insights into the roles of the amphid sensory neurons gained in our study could be extended in many ways to understand pathogen recognition. Going forward, these neurons can be examined for the presence of canonical and non-canonical PRRs. This can be facilitated using the single-cell RNASeq data available for *C. elegans* neurons, including those in the amphid sensilla (Taylor et al, 2021). This needs to go hand in hand with the identification of classical and non-canonical molecular patterns from natural microbiota that *C. elegans* encounters in nature. Studies on neuronal sensing of microbes in *C. elegans* are likely to provide better insight into neuro-immune regulations of animal health in general.

## Materials and Methods

### *C. elegans* strains and maintenance

All strains of *C. elegans* were maintained at 20°C as hermaphrodites on nematode growth media plates seeded with *E. coli* OP50, as previously described (Brenner, 1974). The Bristol N2 strain of *C. elegans* was used as the WT in all experiments. All strains used in this study are listed in Table S3, along with their source and backcross status.

Table S3. This table contains the details of all the strains of C. elegans used in this study.

### Bacterial strains and growth

*P*. *aeruginosa* PA14 was grown on LB broth overnight at 37°C and 50 μl of the overnight culture was spread on slow-killing agar (SK) plates (Tan et al, 1999). The plates were then incubated at 37°C for 12 h for *C. elegans* survival assays. *E*. *faecalis* OG1RF was grown in brain heart infusion (BHI) broth supplemented with 50 μg/ml of gentamycin at 37°C for 8 h and 50 μl of the culture was spread on BHI agar plates supplemented with 50 μg/ml of gentamycin. The plates were then incubated at 37°C for 12 h for *C. elegans* survival assays. *C*. *neoformans* H99α was grown in Yeast-extract Peptone Dextrose broth for 12 h at 25°C and 50 μl of the culture was spread on BHI agar plates. The plates were then incubated at 25°C for an additional 12 h for *C. elegans* survival assays. *E. coli* OP50 was grown in LB broth supplemented with 50 μg/ml of streptomycin at 37°C overnight. 200 μl of the culture was spotted on nematode growth media plates for the maintenance of *C. elegans*.

### Survival assays on pathogens

The survival assays with the three pathogens were performed as previously described (Tan et al, 1999; Garsin et al, 2001; Mylonakis et al, 2002). 50–60 age-synchronized young adults were transferred to the pathogen plates and the plates were incubated at 25°C till the entire population of worms was dead. The plates were scored for live and dead worms twice a day. Kaplan–Meier plots for survival were plotted and analyzed by log-rank test. At least three biological repeats were performed and the statistics for each biological repeat for survival assays are presented in Table S1.

### Quantification of intestinal pathogen load

To enumerate the intestinal load of pathogens, 50 worms were transferred and exposed to individual pathogens (PA14, OG1RF or H99α) at 25°C for 24 h. 15 worms were picked and washed thrice in M9 buffer containing 50 μg/ml gentamycin, 50 μg/ml carbenicillin, and 25 mM levamisole, followed by three washes with plain M9 buffer. The worms were then crushed and the lysate was serially diluted. 100 μl of the 10^−5^ and 10^−6^ dilutions were plated on agar plates with appropriate growth media (cetrimide agar media for PA14, BHI agar with 50 μg/ml gentamycin for OG1RF, and Yeast-extract Peptone Dextrose agar for H99α) and incubated at 37°C for 12–16 h for enumerating PA14 and OG1RF and at 25°C for 24 h for enumerating H99α. The number of CFU per infected worm was calculated and plotted to obtain mean ± SEM. At least three biological replicates were used per genotype per pathogen.

### Quantification of pharyngeal pumping

Age-synchronized young adults were transferred to OP50 plates or the pathogen assay plates and incubated at 25°C for 12 h. After the incubation, the worms were observed under the microscope mounted with a 2x magnification lens and counted for the pumping of the lower pharyngeal bulb. Videos were made for 15 worms for 30 s each using a Nikon D3500 camera, and the pumping rate was calculated by counting the number of contractions of the terminal pharyngeal bulb for 30 s. The pharyngeal pumps per minute were then plotted as violin plots to obtain mean ± SEM. Three biological replicates of 15 worms each were used per genotype for OP50 or individual pathogens.

### Generation of amphid neuron ablation lines

**Figure S4.**
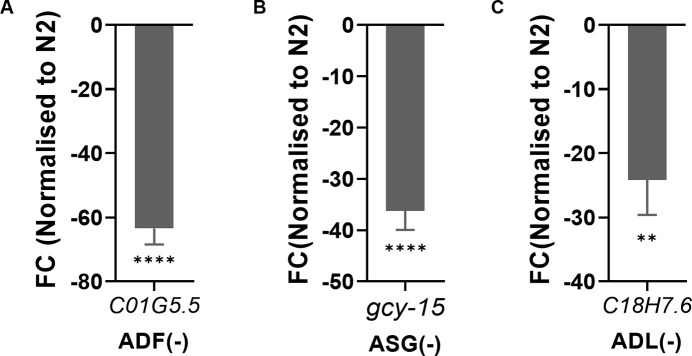
Validating neuronal ablation lines through qRT–PCR. **(A, B, C)** Expression levels of neuron-specific transcripts quantified by qRT–PCR for (A) ADF(−), (B) ASG(−), and (C) ADL(−). Significance was calculated using unpaired *t* test. ****, *P* < 0.0001; **. *P* < 0.01. Source data are available for this figure.

The neuronal ablation lines used in this study were generated using the expression of the split recCaspase system (Chelur & Chalfie, 2007) using cell-specific promoters. Promoter sequences of *tph-1* and *ntr-1* were cloned into TU#813 and TU#814, respectively, for making the ADF(−) line. For the generation of the ASG(−) line, the *gcy-15* promoter was cloned into the recCaspase plasmids. The *srh-220* promoter sequences were cloned into TU#813 and TU#814 for the ADL(−) line. 25 ng/μl each of TU#813 and TU#814 containing the cell-specific promoters upstream of the recCaspase was injected into gravid N2 worms, along with 50 ng/μl of the *unc122*p::GFP plasmid for selection. The ablations of amphid neurons generated in this study were confirmed by qRT–PCR of neuron-specific transcript for ablation strains created in this study (Fig S4). Neuron-specific transcripts were identified using the CeNGEN database with a cut-off of two or above (Taylor et al, 2021). Primer sequences for cloning specific promoters and qRT PCR of neuron-specific transcripts are available upon request.

**Figure S5.**
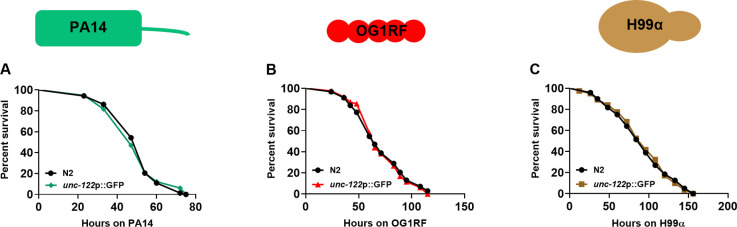
*unc*-122p::GFP worms survive similar to WT upon infection. **(A, B, C)** Kaplan–Meier survival curves for N2 and *unc:122*p::GFP on (A) PA14 (*P* = 0.8111), (B) OG1RF (*P* = 0.817), and (C) H99α (*P* = 0.9454).

Because the caspase-based ablation lines used *unc-122*p::GFP as a co-marker, we created *unc-122*p::GFP alone as a control. The survival phenotype of *unc-122*p::GFP animals was comparable with that of N2 WT on all three pathogens (Fig S5).

### RNA extraction, cDNA synthesis, and qRT–PCR

RNA was extracted from 1,000–1,200 age-synchronized L4 worms using RNeasy Plus Universal Mini kit (Cat. No. 73404; QIAGEN) as per the manufacturer’s protocol. cDNA was prepared using the iScript cDNA synthesis kit (Cat. No. 1708891; BIO-RAD) as per the manufacturer’s protocol. qRT–PCR was performed using the iTaq Universal SYBR Green mix (Cat. No. 172-5124; BIO-RAD) on an Applied Biosystems QuantStudio three real-time PCR machine in a 96-well plate format. Fifty nanograms of cDNA were used for qRT–PCR. 10 μl reactions were set-up in two technical replicates and performed as outlined by the manufacturer. Relative-fold changes were calculated using the comparative CT (2^−∆∆CT^) method and normalized to *act-1* (Livak & Schmittgen, 2001).

### Statistical analysis

Survival graphs were analyzed using the GraphPad PRISM 5.01 software (Kaplan–Meier method). Survival curves with *P*-values < 0.05 using the Mental-Cox log-rank test were considered significantly different. Survival statistics are presented in Table S1. For analyses of pharyngeal pumping rates (Fig S2), post-hoc Dunnett’s test was used to compare each genotype with WT N2. For analyses of intestinal load of pathogens, post-hoc Dunnett’s test was used to compare each genotype with WT N2 for each pathogen. For analyses of qRT–PCR data, unpaired *t* test was used to compare neuron-specific transcript levels of individual ablation lines with WT N2.

## Supplementary Material

Reviewer comments
