# Supplementary material for: Amphid sensory neurons of Caenorhabditis elegans orchestrate its survival from infection with broad classes of pathogens
Source: Life Sci Alliance. 2023 May 31;6(8):e202301949. doi: 10.26508/lsa.202301949 (PMC10233725; doi:10.26508/lsa.202301949)
Supplement: Supplementary file 2 [file LSA-2023-01949_TableS1.docx]

**Amphid sensory neurons of *Caenorhabditis elegans* orchestrate its survival from infection with broad classes of pathogens**

Siddharth R Venkatesh, Anjali Gupta, Varsha Singh

Correspondence: [varsha@iisc.ac.in](mailto:varsha@iisc.ac.in)

**Supplementary Table S1A:**

| **Pathogen** | **Genotype** | ***Number of animals (Assayed/Censored)*** | **TD_50_ in Hours** | **P-value** | **Figure** |
| --- | --- | --- | --- | --- | --- |
| **PA14** | N2  *odr-7* | 115/33  125/34 | 62.7  60.8 | P = 0.126 | 1A |
|  | N2  *odr-7* | 135/32  110/25 | 58.3  61.8 | P = 0.0067 |  |
|  | N2  *odr-7* | 140/54  135/35 | 51.7  54 | P = 0.2721 |  |
|  | N2  AWA(-) [agEx[odr-10p::TU#813 + odr-10p::TU#814 + unc-122p::GFP]] | 123/21  110/23 | 48.5  48.7 | P = 0.1288 | S1A |
|  | N2  AWA(-) [agEx[odr-10p::TU#813 + odr-10p::TU#814 + unc-122p::GFP]] | 125/8  125/8 | 44.5  44.5 | P = 0.6194 |  |
|  | N2  AWA(-) [agEx[odr-10p::TU#813 + odr-10p::TU#814 + unc-122p::GFP]] | 100/11  125/6 | 48.2  48.6 | P = 0.2944 |  |
| **PA14** | N2  *lim-4(yz12)* | 135/32  110/24 | 58.3  53.3 | P < 0.0001 |  |
|  | N2  *lim-4(yz12)* | 130/37  110/13 | 57.9  50.2 | P < 0.0001 | 1D |
|  | N2  *lim-4(yz12)* | 140/34  97/18 | 56.5  48.8 | P < 0.0001 |  |
|  |  |  |  |  |  |
| **PA14** | N2  AWC(-) [oyIs85 [*ceh-36*p::TU#813 + *ceh*-36p::TU#814 + *srtx-1*p::GFP + *unc-122*p::DsRed]] | 135/17  120/25 | 43.5  56.2 | P < 0.0001 | 1G |
|  | N2  AWC(-) [oyIs85 [*ceh-36*p::TU#813 + *ceh*-36p::TU#814 + *srtx-1*p::GFP + *unc-122*p::DsRed]] | 135/17  120/25 | 43.7  54.9 | P < 0.0001 |  |
|  | N2  AWC(-) [oyIs85 [*ceh-36*p::TU#813 + *ceh*-36p::TU#814 + *srtx-1*p::GFP + *unc-122*p::DsRed]] | 109/24  108/21 | 43.8  52.7 | P = 0.0003 |  |
| **PA14** | N2  *gcy-8(oy44)* | 132/14  116/24 | 42.0  36.9 | P < 0.0001 | 2A |
|  | N2  *gcy-8(oy44)* | 116/10  107/3 | 44.9  39.1 | P = 0.05 |  |
|  | N2  *gcy-8(oy44)* | 107/42  93/18 | 48.1  43.4 | P = 0.02 |  |
|  | N2  AFD(-) [pgIS1 [gcy-8p::TU#813 + gcy-8p::TU#814 + unc-122p::GFP + gcy-8p::mCherry + gcy-8p::GFP + ttx-3p::GFP]] | 132/14  130/22 | 42.0  37.4 | P < 0.0001 | S1D |
|  | N2  AFD(-) [pgIS1 [gcy-8p::TU#813 + gcy-8p::TU#814 + unc-122p::GFP + gcy-8p::mCherry + gcy-8p::GFP + ttx-3p::GFP]] | 107/42  123/19 | 47.8  41.2 | P = 0.0005 |  |
|  | N2  AFD(-) [pgIS1 [gcy-8p::TU#813 + gcy-8p::TU#814 + unc-122p::GFP + gcy-8p::mCherry + gcy-8p::GFP + ttx-3p::GFP]] | 121/41  110/4 | 47.9  44.1 | P = 0.0019 |  |
| **PA14** | N2  *che-1(p672)* | 135/17  101/21 | 43.5  54.8 | P < 0.0001 | S1J |
|  | N2  *che-1(p672)* | 109/24  119/17 | 43.8  50.2 | P = 0.0024 |  |
|  | N2  *che-1(p672)* | 125/31  130/18 | 50.7  56.9 | P = 0.0008 |  |
| **PA14** | N2  ADF(-) [agEx[*tph-1*p::TU#813 + *ntr-1*p::TU#814 + *unc-122*p::GFP]] | 120/4  121/7 | 48.6  42.8 | P = 0.0004 | 2D |
|  | N2  ADF(-) [agEx[*tph-1*p::TU#813 + *ntr-1*p::TU#814 + *unc-122*p::GFP]] | 120/3  118/1 | 48.6  41.8 | P = 0.0024 |  |
|  | N2  ADF(-) [agEx[*tph-1*p::TU#813 + *ntr-1*p::TU#814 + *unc-122*p::GFP]] | 121/0  119/1 | 47  42.5 | P = 0.0357 |  |
| **PA14** | N2  ASG(-) [asEx[*gcy-15*p::TU#813 + *gcy-15*p::TU#814 + *unc-122*p::GFP]] | 123/21  98/25 | 47.8  51.1 | P = 0.0030 | 3A |
|  | N2  ASG(-) [asEx[*gcy-15*p::TU#813 + *gcy-15*p::TU#814 + *unc-122*p::GFP]] | 100/11  84/7 | 48.7  54.4 | P = 0.0039 |  |
|  | N2  ASG(-) [asEx[*gcy-15*p::TU#813 + *gcy-15*p::TU#814 + *unc-122*p::GFP]] | 125/8  149/21 | 44.4  48.9 | P = 0.0069 |  |
| **PA14**  **PA14** | N2  ASH(-) [agEx[*sra-6*p::TU#813 + *del-2*p::TU#814 + *unc-122*p::GFP]] | 123/21  107/14 | 47.8  41.3 | P < 0.0001 | S1G |
|  | N2  ASH(-) [agEx[*sra-6*p::TU#813 + *del-2*p::TU#814 + *unc-122*p::GFP]] | 100/11  91/13 | 48.6  40.9 | P = 0.0110 |  |
|  | N2  ASH(-) [agEx[*sra-6*p::TU#813 + *del-2*p::TU#814 + *unc-122*p::GFP]] | 120/4  121/9 | 48.6  42.9 | P = 0.0003 |  |
|  | N2  pqe-1;rtIs11 [osm-10p::GFP + osm-10p::HtnQ150 + dpy-20(+)] | 88/8  122/6 | 42.7  36.6 | P < 0.0001 | 2G |
|  | N2  pqe-1;rtIs11 [osm-10p::GFP + osm-10p::HtnQ150 + dpy-20(+)] | 80/7  80/9 | 38.5  35.7 | P = 0.054 |  |
|  | N2  pqe-1;rtIs11 [osm-10p::GFP + osm-10p::HtnQ150 + dpy-20(+)] | 125/31  105/46 | 50.6  41.9 | P = 0.0013 |  |
| **PA14** | N2  ASI(-) [oyIs84 [gpa-4p::TU#813 + gcy-27p::TU#814 + gcy-27p::GFP + unc-122p::DsRed]] | 125/8  120/16 | 44.4  48.7 | P = 0.0001 | 3D |
|  | N2  ASI(-) [oyIs84 [gpa-4p::TU#813 + gcy-27p::TU#814 + gcy-27p::GFP + unc-122p::DsRed]] | 109/24  123/33 | 44  53.4 | P = 0.0003 |  |
|  | N2  ASI(-) [oyIs84 [gpa-4p::TU#813 + gcy-27p::TU#814 + gcy-27p::GFP + unc-122p::DsRed]] | 135/17  112/4 | 43.6  59.7 | P < 0.0001 |  |
| **PA14**  **PA14** | N2  *trx-1(ok1449)* | 132/14  140/24 | 41.3  42.2 | P = 0.8917 |  |
|  | N2  *trx-1(ok1449)* | 121/41  145/8 | 47.8  52.1 | P = 0.4388 |  |
|  | N2  *trx-1(ok1449)* | 107/42  121/32 | 48  47.1 | P = 0.9437 | S1M |
| **PA14** | N2  ASK(-) qrIS1 [sra-9::mCasp1] | 130/37  145/38 | 57.4  69.7 | P < 0.0001 | 3G |
|  | N2  ASK(-) qrIS1 [sra-9::mCasp1] | 135/32  145/43 | 58.2  68.3 | P < 0.0001 |  |
|  | N2  ASK(-) qrIS1 [sra-9::mCasp1] | 140/54  140/25 | 51.6  71.8 | P < 0.0001 |  |
| **PA14** | N2  ADL(-) [asEx[*srh-220*p::TU#813 + *srh-220*p::TU#814 + *unc-122*p::GFP]] | 121/0  120/0 | 47.2  59.7 | P = 0.0004 | S1P |
|  | N2  ADL(-) [asEx[*srh-220*p::TU#813 + *srh-220*p::TU#814 + *unc-122*p::GFP]] | 120/4  120/12 | 48.7  60.1 | P < 0.0001 |  |
|  | N2  ADL(-) [asEx[*srh-220*p::TU#813 + *srh-220*p::TU#814 + *unc-122*p::GFP]] | 120/3  125/3 | 48.6  55.4 | P = 0.0004 |  |
| **PA14** | N2  agEx[*unc-122*p::GFP] | 120/4  122/3 | 48.6  47.9 | P = 0.5553 |  |
|  | N2  agEx[*unc-122*p::GFP] | 123/21  115/10 | 47.8  45.7 | P = 0.8111 | S4A |
|  | N2  agEx[*unc-122*p::GFP] | 120/3  122/4 | 48.6  50.2 | P = 0.3338 |  |

**Supplementary Table S1B:**

| **Pathogen** | **Genotype** | **Number of animals (Assayed/Censored)** | **TD_50_ in Hours** | **P-value** | **Figure** |
| --- | --- | --- | --- | --- | --- |
| **OG1RF** | N2  *odr-7* | 136/39  130/20 | 77.4  90.9 | P < 0.0001 | 1B |
|  | N2  *odr-7* | 125/1  120/1 | 63.8  78.9 | P < 0.0001 |  |
|  | N2  *odr-7* | 112/12  113/22 | 64.6  73 | P = 0.0082 |  |
|  | N2  AWA(-) [agEx[odr-10p::TU#813 + odr-10p::TU#814 + unc-122p::GFP]] | 122/10  127/13 | 64.6  74.8 | P = 0.0111 | S1B |
|  | N2  AWA(-) [agEx[odr-10p::TU#813 + odr-10p::TU#814 + unc-122p::GFP]] | 125/3  125/17 | 66.1  77.8 | P = 0.0062 |  |
|  | N2  AWA(-) [agEx[odr-10p::TU#813 + odr-10p::TU#814 + unc-122p::GFP]] | 130/27  125/7 | 63.2  71.2 | P < 0.0001 |  |
| **OG1RF** | N2  *lim-4(yz12)* | 126/45  135/23 | 70.8  72.4 | P = 0.5014 | 1E |
|  | N2  *lim-4(yz12)* | 136/39  140/65 | 77.4  79.2 | P = 0.5320 |  |
|  | N2  *lim-4(yz12)* | 161/42  156/39 | 61.2  66.3 | P = 0.1889 |  |
| **OG1RF** | N2  AWC(-) [oyIs85 [*ceh-36*p::TU#813 + *ceh*-36p::TU#814 + *srtx-1*p::GFP + *unc-122*p::DsRed]] | 112/12  137/20 | 64.5  71 | P = 0.0041 | 1H |
|  | N2  AWC(-) [oyIs85 [*ceh-36*p::TU#813 + *ceh*-36p::TU#814 + *srtx-1*p::GFP + *unc-122*p::DsRed]] | 126/45  124/31 | 70.8  80.9 | P = 0.0018 |  |
|  | N2  AWC(-) [oyIs85 [*ceh-36*p::TU#813 + *ceh*-36p::TU#814 + *srtx-1*p::GFP + *unc-122*p::DsRed]] | 136/39  120/30 | 77.4  91.7 | P < 0.0001 |  |
| **OG1RF** | N2  *gcy-8(oy44)* | 105/9  120/14 | 59.9  54.9 | P = 0.0020 |  |
|  | N2  *gcy-8(oy44)* | 124/12  60/5 | 74.3  59.4 | P < 0.0001 |  |
|  | N2  *gcy-8(oy44)* | 130/22  115/20 | 65.3  48.4 | P < 0.0001 | 2B |
|  | N2  AFD(-) [pgIS1 [gcy-8p::TU#813 + gcy-8p::TU#814 + unc-122p::GFP + gcy-8p::mCherry + gcy-8p::GFP + ttx-3p::GFP]] | 124/12  129/21 | 74.3  54.8 | P < 0.0001 |  |
|  | N2  AFD(-) [pgIS1 [gcy-8p::TU#813 + gcy-8p::TU#814 + unc-122p::GFP + gcy-8p::mCherry + gcy-8p::GFP + ttx-3p::GFP]] | 130/22  115/15 | 65.3  54.4 | P < 0.0001 | S1E |
|  | N2  AFD(-) [pgIS1 [gcy-8p::TU#813 + gcy-8p::TU#814 + unc-122p::GFP + gcy-8p::mCherry + gcy-8p::GFP + ttx-3p::GFP]] | 105/9  115/6 | 59.9  48.8 | P = 0.0062 |  |
| **OG1RF** | N2  *che-1(p672)* | 125/1  109/0 | 63.8  65 | P = 0.7011 |  |
|  | N2  *che-1(p672)* | 112/12  135/14 | 64.6  66.9 | P = 0.3239 | S1K |
|  | N2  *che-1(p672)* | 122/12  125/8 | 64.3  67.9 | P = 0.0982 |  |
| **OG1RF** | N2  ADF(-) [agEx[*tph-1*p::TU#813 + *ntr-1*p::TU#814 + *unc-122*p::GFP]] | 120/5  115/6 | 66  56.4 | P = 0.0035 | 2E |
|  | N2  ADF(-) [agEx[*tph-1*p::TU#813 + *ntr-1*p::TU#814 + *unc-122*p::GFP]] | 125/3  120/10 | 66.1  61.9 | P = 0.0100 |  |
|  | N2  ADF(-) [agEx[*tph-1*p::TU#813 + *ntr-1*p::TU#814 + *unc-122*p::GFP]] | 112/10  120/8 | 64.5  60.5 | P = 0.0002 |  |
| **OG1RF** | N2  ASG(-) [asEx[*gcy-15*p::TU#813 + *gcy-15*p::TU#814 + *unc-122*p::GFP]] | 130/27  120/20 | 63.2  71.3 | P < 0.0001 |  |
|  | N2  ASG(-) [asEx[*gcy-15*p::TU#813 + *gcy-15*p::TU#814 + *unc-122*p::GFP]] | 120/4  125/5 | 60.3  68.3 | P = 0.0050 | 3B |
|  | N2  ASG(-) [asEx[*gcy-15*p::TU#813 + *gcy-15*p::TU#814 + *unc-122*p::GFP]] | 113/17  115/29 | 60.6  67.2 | P = 0.0326 |  |
| **OG1RF**  **OG1RF** | N2  ASH(-) [agEx[*sra-6*p::TU#813 + *del-2*p::TU#814 + *unc-122*p::GFP]] | 130/27  123/9 | 63.2  53.3 | P = 0310 |  |
|  | N2  ASH(-) [agEx[*sra-6*p::TU#813 + *del-2*p::TU#814 + *unc-122*p::GFP]] | 120/4  120/4 | 60.7  57.8 | P = 0.038 | S1H |
|  | N2  ASH(-) [agEx[*sra-6*p::TU#813 + *del-2*p::TU#814 + *unc-122*p::GFP]] | 113/17  120/17 | 60.6  59.5 | P = 0.0296 |  |
|  | N2  pqe-1;rtIs11 [osm-10p::GFP + osm-10p::HtnQ150 + dpy-20(+)] | 122/12  119/11 | 64.3  58.8 | P <0.0001 | 2H |
|  | N2  pqe-1;rtIs11 [osm-10p::GFP + osm-10p::HtnQ150 + dpy-20(+)] | 125/13  122/6 | 62.8  57 | P = 0.0053 |  |
|  | N2  pqe-1;rtIs11 [osm-10p::GFP + osm-10p::HtnQ150 + dpy-20(+)] | 122/0  118/5 | 66  62.3 | P < 0.0001 |  |
| **OG1RF** | N2  ASI(-) [oyIs84 [gpa-4p::TU#813 + gcy-27p::TU#814 + gcy-27p::GFP + unc-122p::DsRed]] | 130/27  120/9 | 63.2  77.9 | P < 0.0001 |  |
|  | N2  ASI(-) [oyIs84 [gpa-4p::TU#813 + gcy-27p::TU#814 + gcy-27p::GFP + unc-122p::DsRed]] | 112/12  110/23 | 64.3  77.5 | P = 0.0004 | 3E |
|  | N2  ASI(-) [oyIs84 [gpa-4p::TU#813 + gcy-27p::TU#814 + gcy-27p::GFP + unc-122p::DsRed]] | 113/17  120/13 | 60.6  68.4 | P = 0.0326 |  |
| **OG1RF**  **OG1RF** | N2  *trx-1(ok1449)* | 127/10  127/19 | 66.9  70.8 | P = 0.0548 |  |
|  | N2  *trx-1(ok1449)* | 130/22  105/24 | 65.3  67.6 | P = 0.6336 | S1N |
|  | N2  *trx-1(ok1449)* | 124/14  101/33 | 74.6  70.8 | P = 0.4994 |  |
| **OG1RF** | N2  ASK(-) qrIS1 [sra-9::mCasp1] | 161/42  145/37 | 61.2  82 | P < 0.0001 |  |
|  | N2  ASK(-) qrIS1 [sra-9::mCasp1] | 112/4  140/19 | 52.5  79.8 | P < 0.0001 | 3H |
|  | N2  ASK(-) qrIS1 [sra-9::mCasp1] | 125/23  145/43 | 57.3  70.1 | P = 0.0005 |  |
| **OG1RF** | N2  ADL(-) [asEx[*srh-220*p::TU#813 + *srh-220*p::TU#814 + *unc-122*p::GFP]] | 125/13  117/13 | 62.8  75.9 | P = 0.0009 |  |
|  | N2  ADL(-) [asEx[*srh-220*p::TU#813 + *srh-220*p::TU#814 + *unc-122*p::GFP]] | 120/5  125/8 | 66  70.3 | P = 0.0318 | S1Q |
|  | N2  ADL(-) [asEx[*srh-220*p::TU#813 + *srh-220*p::TU#814 + *unc-122*p::GFP]] | 122/0  120/3 | 66  70.3 | P = 0.0229 |  |
| **OG1RF** | N2  agEx[*unc-122*p::GFP] | 125/13  123/4 | 62.8  63.4 | P = 0.817 | S4B |
|  | N2  agEx[*unc-122*p::GFP] | 122/10  122/10 | 64.6  64.9 | P = 0.4313 |  |
|  | N2  agEx[*unc-122*p::GFP] | 125/3  123/23 | 66.1  65.1 | P = 0.0958 |  |

**Supplementary Table 1C:**

| **Pathogen** | **Genotype** | **Number of animals (Assayed/Censored)** | **TD_50_ in Hours** | **P-value** | **Figure** |
| --- | --- | --- | --- | --- | --- |
| **H99**α | N2  *odr-7* | 110/12  105/18 | 85  75.2 | P = 0.0011 | 1C |
|  | N2  *odr-7* | 130.29  132/20 | 96.1  67.4 | P < 0.0001 |  |
|  | N2  *odr-7* | 120/32  123/58 | 83.8  73.2 | P = 0.0005 |  |
|  | N2  AWA(-) [agEx[odr-10p::TU#813 + odr-10p::TU#814 + unc-122p::GFP]] | 120/26  123/18 | 84.7  69.7 | P = 0.0012 | S1C |
|  | N2  AWA(-) [agEx[odr-10p::TU#813 + odr-10p::TU#814 + unc-122p::GFP]] | 122/23  119/18 | 88.6  72 | P = 0.0110 |  |
|  | N2  AWA(-) [agEx[odr-10p::TU#813 + odr-10p::TU#814 + unc-122p::GFP]] | 127/18  123/12 | 83  69.1 | P = 0.0012 |  |
| **H99**α | N2  *lim-4(yz12)* | 130/29  122/47 | 96.1  96.44 | P = 0.4655 | 1F |
|  | N2  *lim-4(yz12)* | 122/23  120/21 | 88.6  93.4 | P = 0.4039 |  |
|  | N2  *lim-4(yz12)* | 127/18  128/13 | 83  85.1 | P = 0.9703 |  |
| **H99**α | N2  AWC(-) [oyIs85 [*ceh-36*p::TU#813 + *ceh*-36p::TU#814 + *srtx-1*p::GFP + *unc-122*p::DsRed]] | 130/29  155/47 | 96.1  122 | P < 0.0001 | 1I |
|  | N2  AWC(-) [oyIs85 [*ceh-36*p::TU#813 + *ceh*-36p::TU#814 + *srtx-1*p::GFP + *unc-122*p::DsRed]] | 110/12  91/26 | 84.7  94.4 | P = 0.0006 |  |
|  | N2  AWC(-) [oyIs85 [*ceh-36*p::TU#813 + *ceh*-36p::TU#814 + *srtx-1*p::GFP + *unc-122*p::DsRed]] | 120/26  128/36 | 84.7  112.3 | P < 0.0001 |  |
| **H99**α | N2  *gcy-8(oy44)* | 114/54  90/7 | 87.8  77.8 | P = 0.0071 |  |
|  | N2  *gcy-8(oy44)* | 100/33  110/26 | 82.4  68.9 | P = 0.0002 | 2C |
|  | N2  *gcy-8(oy44)* | 127/18  115/15 | 83  67 | P = 0.0017 |  |
|  | N2  AFD(-) [pgIS1 [gcy-8p::TU#813 + gcy-8p::TU#814 + unc-122p::GFP + gcy-8p::mCherry + gcy-8p::GFP + ttx-3p::GFP]] | 114/54  80/11 | 87.8  64.7 | P < 0.0001 |  |
|  | N2  AFD(-) [pgIS1 [gcy-8p::TU#813 + gcy-8p::TU#814 + unc-122p::GFP + gcy-8p::mCherry + gcy-8p::GFP + ttx-3p::GFP]] | 100/33  128/46 | 82.4  67.3 | P = 0.0002 | S1F |
|  | N2  AFD(-) [pgIS1 [gcy-8p::TU#813 + gcy-8p::TU#814 + unc-122p::GFP + gcy-8p::mCherry + gcy-8p::GFP + ttx-3p::GFP]] | 120/32  60/10 | 83.8  70.8 | P = 0.0019 |  |
| **H99**α | N2  *che-1(p672)* | 124/29  123/27 | 87.7  98.2 | P = 0.0042 | S1L |
|  | N2  *che-1(p672)* | 127/18  110/13 | 83  99.6 | P = 0.0048 |  |
|  | N2  *che-1(p672)* | 127/25  122/20 | 88.1  110.3 | P = 0.0014 |  |
| **H99**α | N2  ADF(-) [agEx[*tph-1*p::TU#813 + *ntr-1*p::TU#814 + *unc-122*p::GFP]] | 120/1  121/14 | 85.3  73.4 | P = 0.0019 | 2F |
|  | N2  ADF(-) [agEx[*tph-1*p::TU#813 + *ntr-1*p::TU#814 + *unc-122*p::GFP]] | 120/26  121/20 | 84.7  67.9 | P = 0.0004 |  |
|  | N2  ADF(-) [agEx[*tph-1*p::TU#813 + *ntr-1*p::TU#814 + *unc-122*p::GFP]] | 122/23  122/16 | 88.6  65.9 | P = 0.0004 |  |
| **H99**α | N2  ASG(-) [asEx[*gcy-15*p::TU#813 + *gcy-15*p::TU#814 + *unc-122*p::GFP]] | 120/1  125/25 | 85.3  99.8 | P = 0.0024 | 3C |
|  | N2  ASG(-) [asEx[*gcy-15*p::TU#813 + *gcy-15*p::TU#814 + *unc-122*p::GFP]] | 120/26  123/28 | 84.7  95.7 | P = 0.0434 |  |
|  | N2  ASG(-) [asEx[*gcy-15*p::TU#813 + *gcy-15*p::TU#814 + *unc-122*p::GFP]] | 122/23  120/34 | 88.6  105.8 | P = 0.0026 |  |
| **H99**α  **H99**α | N2  ASH(-) [agEx[*sra-6*p::TU#813 + *del-2*p::TU#814 + *unc-122*p::GFP]] | 120/26  130/12 | 84.7  65.2 | P < 0.0001 |  |
|  | N2  ASH(-) [agEx[*sra-6*p::TU#813 + *del-2*p::TU#814 + *unc-122*p::GFP]] | 122/23  120/13 | 88.6  67.1 | P = 0.0022 | S1I |
|  | N2  ASH(-) [agEx[*sra-6*p::TU#813 + *del-2*p::TU#814 + *unc-122*p::GFP]] | 127/18  126/20 | 83  65.7 | P = 0.0006 |  |
|  | N2  pqe-1;rtIs11 [osm-10p::GFP + osm-10p::HtnQ150 + dpy-20(+)] | 120/32  102/23 | 83.8  71.33 | P = 0.0074 |  |
|  | N2  pqe-1;rtIs11 [osm-10p::GFP + osm-10p::HtnQ150 + dpy-20(+)] | 100/33  129/32 | 82.9  67.4 | P = 0.0043 | 2I |
|  | N2  pqe-1;rtIs11 [osm-10p::GFP + osm-10p::HtnQ150 + dpy-20(+)] | 127/18  122/19 | 83  67.9 | P = 0.0043 |  |
| **H99**α | N2  ASI(-) [oyIs84 [gpa-4p::TU#813 + gcy-27p::TU#814 + gcy-27p::GFP + unc-122p::DsRed]] | 100/33  117/49 | 82.9  89 | P = 0.0492 | 3F |
|  | N2  ASI(-) [oyIs84 [gpa-4p::TU#813 + gcy-27p::TU#814 + gcy-27p::GFP + unc-122p::DsRed]] | 120/26  124/29 | 84.7  107.6 | P < 0.0001 |  |
|  | N2  ASI(-) [oyIs84 [gpa-4p::TU#813 + gcy-27p::TU#814 + gcy-27p::GFP + unc-122p::DsRed]] | 122/23  124/29 | 88.6  111.2 | P = 0.0002 |  |
| **H99**α  **H99**α | N2  *trx-1(ok1449)* | 114/54  121/35 | 87.8  103.3 | P = 0.0008 |  |
|  | N2  *trx-1(ok1449)* | 124/25  120/45 | 87.9  108.4 | P = 0.0001 | S1O |
|  | N2  *trx-1(ok1449)* | 100/33  119/42 | 82.9  106.7 | P < 0.0001 |  |
| **H99**α | N2  ASK(-) qrIS1 [sra-9::mCasp1] | 130/29  135/51 | 96.1  121 | P < 0.0001 |  |
|  | N2  ASK(-) qrIS1 [sra-9::mCasp1] | 124/29  121/27 | 87.9  107.6 | P < 0.0001 | 3I |
|  | N2  ASK(-) qrIS1 [sra-9::mCasp1] | 115/30  111/29 | 91.4  115.4 | P < 0.0001 |  |
| **H99**α | N2  ADL(-) [asEx[*srh-220*p::TU#813 + *srh-220*p::TU#814 + *unc-122*p::GFP]] | 120/1  120/7 | 85.3  96.3 | P = 0.0042 | S1R |
|  | N2  ADL(-) [asEx[*srh-220*p::TU#813 + *srh-220*p::TU#814 + *unc-122*p::GFP]] | 120/26  122/29 | 84.7  107.8 | P < 0.0001 |  |
|  | N2  ADL(-) [asEx[*srh-220*p::TU#813 + *srh-220*p::TU#814 + *unc-122*p::GFP]] | 122/23  120/19 | 88.6  108.9 | P = 0.0006 |  |
| **H99**α | N2  agEx[*unc-122*p::GFP] | 120/1  120/2 | 85.3  86 | P = 0.9454 | S4C |
|  | N2  agEx[*unc-122*p::GFP] | 127/18  126/20 | 83  86.6 | P = 0.3194 |  |
|  | N2  agEx[*unc-122*p::GFP] | 127/25  125/23 | 88.1  90.8 | P = 0.4744 |  |

**Supplementary Table S1: Survival curve statistics -** This table contains the TD_50_ values on (A) PA14, (B) OG1RF and (C) H99α, number of animals assayed/censored, P-values, and the figures corresponding to the statistics for each of the survival experiments performed for each of the strains used in this study. The statistics were obtained by plotting a Kaplan-Meier plot and the P values were calculated using Log-rank test.
