# Supplementary material for: Amphid sensory neurons of Caenorhabditis elegans orchestrate its survival from infection with broad classes of pathogens
Source: Life Sci Alliance. 2023 May 31;6(8):e202301949. doi: 10.26508/lsa.202301949 (PMC10233725; doi:10.26508/lsa.202301949)
Supplement: Supplementary file 3 [file LSA-2023-01949_TableS2.docx]

**Amphid sensory neurons of *Caenorhabditis elegans* orchestrate its survival from infection with broad classes of pathogens**

Siddharth R Venkatesh, Anjali Gupta, Varsha Singh

Correspondence: varsha@iisc.ac.in

**Supplementary Table S2:**

| Genotype | Neurons affected | Survival on PA14 | | Survival on OG1RF | | Survival on H99α | |
| --- | --- | --- | --- | --- | --- | --- | --- |
|  |  | % TD_50_ w.r.t N2 | P value | % TD_50_ w.r.t N2 | P value | % TD_50_ w.r.t N2 | P value |
| N2 | None | **100 (48.69 ± 0.92 hours)** | - | **100 (65.13 ± 0.73 hours)** | - | **100 (86.36 ± 0.53 hours** | - |
| *odr-7* | AWA | 102.47 | 0.76 | **118.04** | 0.14 | **81.99** | 0.02 |
| *lim-4(yz12)* | AWB | **88.17** | 0.009 | 104.31 | 0.66 | 102.77 | 0.65 |
| oyIs85 [ceh-36p::TU#813 + ceh-36p::TU#814 + srtx-1p::GFP + unc-122p::DsRed] | **AWC** | **125.20** | 0.0003 | **114.27** | 0.21 | **123.66** | 0.07 |
| *gcy-8(oy44)* | **AFD** | **88.39** | 0.1158 | **81.91** | 0.08 | **84.32** | 0.02 |
| *che-1(p672)* | ASE | **117.61** | 0.06 | 103.8 | 0.05 | **119.06** | 0.01 |
| agEx [*tph-1*p::TU#813 + *ntr-1*p::TU#814 + *unc-122*p::GFP] | **ADF** | **88.17** | 0.007 | **91.61** | 0.04 | **80.20** | 0.002 |
| asEx [*gcy-15*p::TU#813 + *gcy-15*p::TU#814 + *unc-122*p::GFP]] | **ASG** | **109.58** | 0.09 | **112.32** | 0.007 | **118.08** | 0.002 |
| agEx[*sra-6*p::TU#813 + *del-2*p::TU#814 + *unc-122*p::GFP] | **ASH** | **86.13** | 0.0007 | **92.58** | 0.08 | **77.29** | 0.0003 |
| *oyIs84* [*gpa-4*p::TU#813+*gcy-27*p::TU#814+*gcy-27*p::GFP+ *unc-122*p::DsRed] | **ASI** | **126.44** | 0.02 | **118.89** | 0.02 | **119.97** | 0.07 |
| *trx-1(ok1449)* | ASJ | 103.1 | 0.71 | 101.42 | 0.80 | **123.23** | 0.0008 |
| *qrIs2*[*sra-9*::mCasp1] | **ASK** | **125.98** | 0.003 | **136.11** | 0.01 | **124.86** | 0.007 |
| asEx[*srh-220*p::TU#813 + *srh-220*p::TU#814 + *unc-122*p::GFP] | **ADL** | **121.29** | 0.002 | **111.30** | 0.02 | **122.61** | 0.008 |

**Supplementary Table S2**: The table summarises the relative percentage of TD_50_ values for mutants or amphid neuronal ablation lines. The percentage change in TD_50_ values mentioned in the table are with respect to N2. Relative TD_50_ percentage, higher than N2 are represented in red and those that are lower than N2 are represented in green. P values were calculated using unpaired *t*-test.
