# Supplementary material for: Amphid sensory neurons of Caenorhabditis elegans orchestrate its survival from infection with broad classes of pathogens
Source: Life Sci Alliance. 2023 May 31;6(8):e202301949. doi: 10.26508/lsa.202301949 (PMC10233725; doi:10.26508/lsa.202301949)
Supplement: Supplementary file 7 [file LSA-2023-01949_TableS3.docx]

**Amphid sensory neurons of *Caenorhabditis elegans* orchestrate its survival from infection with broad classes of pathogens**

Siddharth R Venkatesh, Anjali Gupta, Varsha Singh

Correspondence: varsha@iisc.ac.in

**Supplementary Table S3: *C*. *elegans* strains used in this study**

| **Strain Name** | **Neurons Affected** | **Genotype** | **Outcross status with N2** | **Reference** |
| --- | --- | --- | --- | --- |
| CX4 | **AWA** | ***odr-7(ky04)*** | **x2** | **Sengupta *et al*., 1994** |
| VSL1504 | **AWA** | agEx[*odr-10*p::TU#813 + *odr-10*p::TU#814 + *unc-122*p::GFP] | - | **Prakash *et al*., 2021** |
| JY359 | **AWB** | ***lim-4(yz12)*** | **x2** | **Sagasti *et al*., 1999** |
| PY7502 | **AWC** | oyIs85 [*ceh-36*p::TU#813 + *ceh*-36p::TU#814 + *srtx-1*p::GFP + *unc-122*p::DsRed] | - | **Beverly *et al*., 2011** |
| IK800 | **AFD** | ***gcy-8(oy44)*** | **x4** | **Inada *et al*., 2006** |
| GN112 | **AFD** | pgIs2 [*gcy-8*p::TU#813 + *gcy-8*p::TU#814 + *unc-122*p::GFP + *gcy-8*p::mCherry + *gcy-8*p::GFP + *ttx-3*p::GFP] | x1 | **Wang *et al*., 2013** |
| PR672 | **ASE** | ***che-1(p672)*** | **x2** | **Uchida *et al*., 2003** |
| VSL1612 | **ADF** | agEx[*tph-1*p::TU#813 + *ntr-1*p::TU#814 + *unc-122*p::GFP] | - | **This study** |
| VSL1601 | **ASG** | asEx[*gcy-15*p::TU#813 + *gcy-15*p::TU#814 + *unc-122*p::GFP] | - | **This study** |
| HA759 | **ASH** | *rtIs11*[*osm-10*p::GFP + *osm-10*p::HtnQ150 + *dpy-20*(+)] | - | **Faber *et al*., 1999** |
| VSL1607 | **ASH** | agEx[*sra-6*p::TU#813 + *del-2*p::TU#814 + *unc-122*p::GFP] | - | **Prakash *et al*., 2012** |
| PY7505 | **ASI** | *oyIs84*[*gpa-4*p::TU#813+*gcy-27*p::TU#814+*gcy-27*p::GFP+ *unc-122*p::DsRed] | - | **Beverly *et al*., 2011** |
| VZ1 | **ASJ** | ***trx-1(ok1449)*** | **x6** | **Miranda-Vizuete *et al*., 2006** |
| PS6025 | **ASK** | *qrIs2*[*sra-9*::mCasp1] | - | **Srinivasan *et al*., 2012** |
| VSL1602 | **ADL** | asEx[*srh-220*p::TU#813 + *srh-220*p::TU#814 + *unc-122*p::GFP] | - | **This study** |
| VSL1501 | **-** | agEx[*unc-122*p::GFP] | - | **This study** |

**Supplementary Table S3:** This table contains the details of all the strains of *C*. *elegans* used this study.
